# Early mortality in German patients with lung cancer: risk factors associated with 30-and 60-day mortality

**DOI:** 10.1007/s10238-023-01187-x

**Published:** 2023-09-12

**Authors:** Amanda Tufman, Sophie Schneiderbauer, Julia Walter, Blerina Resuli, Diego Kauffmann-Guerrero, Carlo Mümmler, Pontus Mertsch, Jeremias Götschke, Julia Kovács, Farkhad Manapov, Christian Schneider, Laura Sellmer, Paola Arnold, Volker Heinemann, Jürgen Behr, Daniel Nasseh

**Affiliations:** 1 Department of Medicine V, University Hospital, LMU Munich, Munich, Germany; 2 Comprehensive Cancer Center, University Hospital, LMU Munich, Munich, Germany; 3 Department of Radiation Oncology, University Hospital, LMU Munich, Munich, Germany; 4 Department of Thoracic Surgery Munich, University Hospital, LMU Munich, Munich, Germany; 5 German Center for Lung Research (DZL), Aulweg 130, 35392 Gießen, Germany

**Keywords:** Thoracic cancer, Premature death, Bronchial tumors, Short survival

## Abstract

Despite therapeutic advances, early mortality in lung cancer is still prevalent. In this study, we aimed to assess risk factors for 30- and 60-day mortality in German lung cancer patients. In this retrospective cross-sectional analysis, we used data of lung cancer patients treated at LMU Hospital Munich between 2015 and 2019. We categorized patients into 30-day mortality, 60 day-mortality, and longer survival. We used Student’s t-test and ANOVA to compare means and Chi^2^-test to compare frequencies. We used logistic regression analysis to identify factors associated with a risk for early mortality. Of the 2454 lung cancer patients, 2.0% (*n* = 50) died within 30 and 1.7% (*n* = 41) within 30 to 60 days of diagnosis. Older age and advanced stage at diagnosis were significantly associated with early mortality in the univariate and the multivariate analysis. Patients in the 30-day mortality group significantly more often did not receive tumor-directed therapy. They were also more likely to die in an acute care setting compared to the 60-day mortality group. The group of patients who died unexpectedly (12.0%) was dominantly female, with a high proportion of patients with unintentional weight loss at the time of diagnosis. Our results suggest that in the treatment of patients with lung cancer there is a need for a greater focus on older patients. Moreover, physicians should pay special attention to females with recent weight loss and patients with a comorbidity of diabetes mellitus or renal impairment. Engaging a case manager focused on detecting patients with the above characteristics could help improve overall care.

**Supplementary Information:**

The online version contains supplementary material available at 10.1007/s10238-023-01187-x.

## Introduction

Recent innovations in lung cancer treatment, like novel targeted therapies and immune checkpoint inhibitors, have led to improved long-term survival for patients with lung cancer, especially in advanced stages of disease [1]. However, early mortality occurs in a subset of patients. Identifying factors leading to early mortality can lead to the prevention of premature deaths. Moreover, it could help to identify patients who would benefit from early integration of palliative care to the treatment regimen which can increase survival after diagnosis [2]. Identifying those factors can also improve decision-making and facilitate detecting patients who most likely would not benefit from aggressive therapeutic interventions in advanced stages of the disease.

Several articles on lung cancer patients have identified increasing age [3, 4], advanced stage at diagnosis [4–6], poor performance status [6, 7], and male sex [3, 5], as relevant factors for early mortality. However, the majority of studies only included patients who received either surgical resection or systemic therapy. Additionally, other factors like cause and place of death have not been investigated yet. Especially, cause of death is a relevant indicator on whether a death was preventable or not. Therefore, these factors can give insights to improve patient care.

The main aim of our study was to detect risk factors for 30- and 60-day mortality after initial diagnosis in lung cancer patients. Second, we aimed to describe cause and place of death, as well as previous hospital interaction and comorbidities in patients with 30-day and 60-day mortality.

## Material and methods

### Study population and categorization of early mortality

For this retrospective cross-sectional analysis, we used patient data of the tumor documentation system CREDOS of the LMU Hospital Munich [8]. The dataset consists of structured, manually collected data for all tumor entities, detailing the whole local (in terms of the LMU) disease history centered around a tumor, split into the main categories first-assessment, administered therapies, as well as follow-up (up to 2000 different data fields). The following information was extracted from the registry by utilizing the local analytics framework MOCCA [9]: date of diagnosis, date of birth, date of death or last follow-up, sex, tumor stage, metastases, and histology, as well as therapies and therapy intent of oncological patients. Inclusion criteria were a newly documented diagnosis of a thoracic malignancy, defined as C34* ICD-10-GM, between 2015 and 2019. We excluded patients if the last documented vital sign was within the first 60 days, but no date of death was available. For further analyses, we amended the dataset with information from patient records stored within the hospital information system.

Date of death and date of the last vital sign, within CREDOS, come from different sources: date of last patient movement (e.g., admission, transfer, or administration of therapy), date of in-hospital death or follow-up from external sources (e.g., general practitioner). Based on the time between date of diagnosis and date of death or last vital sign, we categorized the cohort into three main groups, deceased within 30-days, deceased within 31–60 days, and survived for longer than 60 days since diagnosis. A 30- and 60-day survival is an outcome frequently used in analysis of data concerning early mortality in cancer.

The ethics committee of the Ludwig-Maximilians University Munich (reference number 474-16 UE) approved this retrospective study of anonymized data.

### Definition of variables used in the analyses

Our analysis consisted of two parts. In part one; we compared all three groups, regarding variables already available in the dataset (tumor documentation registry/CREDOS). In the second part, we amended the dataset with information found within medical records from the hospital information system, specifically for the 30-day and 60-day mortality groups.

In part one we compared, the following variables across the three mortality groups: age, sex, and tumor stage at diagnosis (using the 7th or 8th Edition of UICC/AJCC TNM staging system, depending on the year of diagnosis), documented distant and cerebral metastases at diagnosis, tumor histology, as well as documented first-line tumor directed therapy (chemo-, immunotherapy, targeted therapy, radiotherapy or surgery) and intent of first-line therapy (curative, palliative, or unknown). In CREDOS, quality of documentation of first-line therapy is not the same for all patients. Therefore, in this study, we only referred to therapies documented in our dataset.

For part two, we extracted the following additional information: cause of death, place of death, previous hospital interaction, and psychological and other comorbidities from medical letters or other clinical documentation. A panel of physicians extracted cause of death from clinical documentation and categorized it as follows:

- Due to infection,
- Cardiac arrest,
- Paraneoplastic,
- Tumor progression
  - Tumor mass,
  - Thoracic (tumor bleeding, retention pneumonia and superior vena cava syndrome)
  - Brain,
- Complication from therapy (chemo-, immune- or targeted therapy, radiotherapy, intraoperative),
- Thrombosis,
- Unexpected after discharge,
- Unknown.

We categorized place of death as either in an acute care setting, which included the intensive care unit (ICU) or intermediate care ward (IMC), and the general ward. The second category was the non-acute care setting, including the palliative ward, home care, or nursing home. The third category included all patients whose place of death was unknown. Previous hospital interaction included information on whether there was ever a contact to a palliative care team (in-hospital as well as ambulatory palliative care team), whether patients were admitted to the emergency room (ER) at diagnosis or between diagnosis and patients’ death, and whether the patient was admitted to the ICU or IMC between diagnosis and death. We included psychological and behavioral factors in the analysis which were defined as:

- Smoking history (current, former, or never smoker),
- Alcohol abuse,
- Psychological diseases (e.g., depression, anxiety disorder).

The comorbidities were:

- Diabetes mellitus,
- Chronic obstructive pulmonary disease (COPD),
- Cardiac disease,
- Renal insufficiency,
- Prior malignancy,
- Apoplexy.

For comparison, body mass index (BMI) was categorized into three groups according to the conventional WHO classification in 2008: underweight (< 18.5 kg/m^2^), normal weight (18.5–24.9 kg/m^2^), and overweight (≥ 25 kg/m^2^).

### Statistical analysis

We calculated means with standard deviation for numerical variables and absolute and relative frequencies for categorical variables in each mortality group. Numerical variables were compared regarding significant differences using t-test for two group comparisons and ANOVA for three-way group comparisons. Relative frequencies were compared using Chi^2^-test and Fisher's exact test when cell numbers were below six. We used multivariate logistic regression models to analyze factors predicting 30- and 60-day mortality. Variables used in this analysis were age, sex, UICC stage and histology. We considered an α level of < 0.05 as significant in all analyses.

### Subgroup and sensitivity analyses

In the second part of our analysis, first we focused on the subgroup of patients with stage IV at diagnosis and second, we excluded the patients who died due to surgical complications. Additionally, we discarded the mortality grouping to compare characteristics and outcomes between patients with and without palliative care interaction, and between patients who died unexpectedly and all others. Here, we used the definition of cause of death and compared patients with unknown cause of death and whose imminent death was unexpected as judged by the panel of physicians, to all other patients.

## Results

### Characteristics of study population stratified by mortality

From 2015 to 2019 2454 patients with thoracic malignancies were treated at LMU hospital and extracted from the tumor documentation registry CREDOS. Fifty patients (2.0%) died within 30 days of diagnosis, 41 (1.7%) within 30–60 days, and 2363 (96.3%) survived for longer than 60 days after diagnosis. Increasing age of patients was significantly associated with shorter survival (*p*-value < 0.0001). The proportion of male patients decreased with increasing survival time, but differences were not significant.

The proportion of patients with stage III or IV disease significantly differed between the mortality groups (*p*-value < 0.001), and was highest in the 60-day mortality group. Additionally, the proportion of patients with distant metastases at time of diagnosis was significantly higher (*p*-value < 0.0001) in this group.

Adenocarcinoma accounted for 53.5% of all patients, followed by 20.2% squamous-cell carcinoma (SCC), 10.6% with small-cell lung cancer (SCLC), 5.9% neuroendocrine tumors, and 9.8% with other histologic subtypes. Histology distribution was not significantly different between the three groups. Table 1 displays patient and tumor characteristics of the whole population stratified by mortality.

**Table 1:** Patient and tumor characteristics

	30 days		31 to 60 days		> 60 days		*p*-value
	Mean	SD	Mean	SD	Mean	SD	
Age in years	70.7	8.96	70.3	8.21	65.3	11.0	< 0.0001
	*n*	%	*n*	%	*n*	%	
*n*	50	2.0	41	1.7	2363	96.3	
*Sex*							
Male	35	70.0	25	61.0	1348	57.0	0.17
Female	15	30.0	16	39.0	1015	43.0	
*Stage at diagnosis*							
Stage I or II	3	6.0	2	4.9	641	27.1	0.001
Stage III or IV	41	82.0	39	95.1	1622	68.6	
Unknown	6	12.0	0	0.0	100	4.2	
*Histology*							
Adenocarcinoma	19	38.0	20	48.8	1275	54.0	0.07
SCLC	7	14.0	4	9.8	249	10.5	0.37
Neuroendocrine tumor	4	8.0	4	9.8	138	5.8	0.71
Squamous-cell carcinoma	13	26.0	13	31.7	474	20.1	0.58
Other	7	14.0	5	12.2	224	9.5	0.42
Distant metastases	36	72.0	35	85.4	1045	44.2	< 0.0001
Cerebral metastases	10	20.0	9	22.0	376	15.9	0.38

Characteristics of patients and extent of disease stratified by mortality. Mortality is categorized as death within 30 days, within 31–60 days, or survival for more than 60 days after first diagnosis of lung cancer.* P*-values derived from ANOVA for age and Chi^2^test for categorical variables

*SCLC* small-cell lung cancer

### Tumor-directed therapy and intent of therapy stratified by mortality

In the 30-day mortality group, 62% of patients did not have any documented tumor-directed therapy. This proportion was significantly higher (*p*-value < 0.0001) than in the 60-day mortality group (34.1%) and in patients who survived longer than 60 days (21.6%). First-line treatment with chemo-, immune- or targeted therapy was significantly more common in the 60-day mortality group (36.6%, *p*-value = 0.03), compared to patients with survival greater than 60 days (30.5%) and the 30-day mortality group (14.0%).

Patients with 30-day mortality received first-line radiotherapy significantly less frequently compared to the other two groups (14.0%, 19.5%, and 28%, *p*-value = 0.05). There was no significant difference in the proportion of patients receiving surgery between groups.

The intent of first therapeutic strategy differed significantly between groups (*p*-value = 0.005). Palliative intent was more frequent in the 60-day mortality group (88.9%), followed by the 30-day mortality group (68.4%) and 47.1% in patients who survived more than 60 days.

### Factors associated with early mortality from multivariate regression analysis

**Fig. 1:**
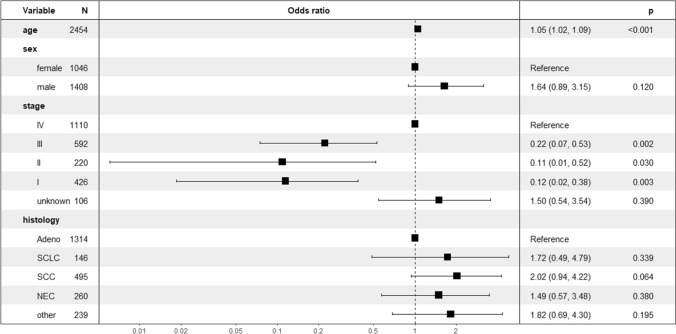
Results from logistic regression analysis of 30-day mortality. Legend: Adeno = adenocarcinoma, SCC = squamous-cell-carcinoma, SCLC = small-cell carcinoma, NEC = neuroendocrine carcinoma

Logistic regression analysis of 30-day mortality showed that age and tumor stage at diagnosis were significantly associated with early mortality. Sex and histological subtype were not significantly associated with survival. Odds Ratios with confidence intervals and p-values for all covariates are displayed in Fig. 1. Logistic regression of 60-day mortality yielded similar results, which are displayed in Fig. 1 of the supplementary material.

### Comparison of cause and place of death, and previous hospital interaction

We did not find any significant differences between patients with 30- and 60-day mortality concerning cause of death. Overall, 49.9% of patients died from tumor progression, which was the most common cause of death in both groups. The second most common cause of death was complications from therapy (16.5%), which occurred intraoperative in 12.1% of patients.

The proportion of patients who died in an acute setting (ICU, IMC or general ward) was significantly higher in the 30-day mortality group compared to the 60-day mortality group (74.0% vs. 41.5%, *p*-value 0.003). Proportions were not significantly different relating to non-acute settings. Significantly more patients in the 30-day mortality group had been admitted to the ICU or IMC between diagnosis and death (42% vs. 14.6%, *p*-value = 0.009). Table 2 summarizes relative frequencies of cause of death, place of death and previous hospital admission stratified by mortality.

**Table 2:** Place of death, cause of death ad hospital interaction stratified by mortality

	30 days (*n* = 50)		31–60 days (*n* = 41)		*p*-value
	*n*	%	*n*	%	
*Cause of death*					
Infection	5	10.0	2	4.9	0.45
Cardiac arrest	4	8.0	1	2.4	1.00
Paraneoplastic	2	4.0	1	2.4	1.00
Tumor progression	24	48.0	21	42.0	0.92
Tumor mass	9	18.0	14	28.0	0.13
Thoracic	9	18.0	6	14.6	0.88
Brain	6	12.0	1	2.4	0.12
Respiratory	3	6.0	1	2.4	0.62
Complication from therapy	9	18.0	6	14.6	0.88
Chemotherapy	*1*	2.0	*2*	4.9	0.59
Radiotherapy	*1*	2.0	*0*	0.0	1.00
Intraoperative	*7*	14.0	*4*	9.8	0.51
Thrombosis	1	2.0	0	0.0	1.00
Unexpected after discharge	4	8.0	7	17.1	0.21
Unknown	1	2.0	2	4.9	0.59
*Place of death*					
Acute setting	37	74.0	17	41.5	0.003
Intensive or intermediate care	13	26.0	5	12.2	0.16
General ward	24	48.0	12	29.3	0.11
Non-acute setting	9	18.0	15	36.6	0.08
Palliative ward	7	14.0	10	24.4	0.32
At home/nursing home	2	4.0	5	12.2	0.24
Unknown	4	8.0	9	22.0	0.11
*Previous hospital interaction*					
Palliative team involved	15	30.0	19	46.3	0.17
Admission to emergency room	15	30.0	14	34.1	0.84
Intensive or intermediate care stay	21	42.0	6	14.6	0.01

Comparison of place of death and cause of death, and hospital interaction of subgroup of patients with 30 and 60-day-mortality. P-values of group comparison from Chi^2^-test

### Psychological and behavioral factors

**Fig. 2:**
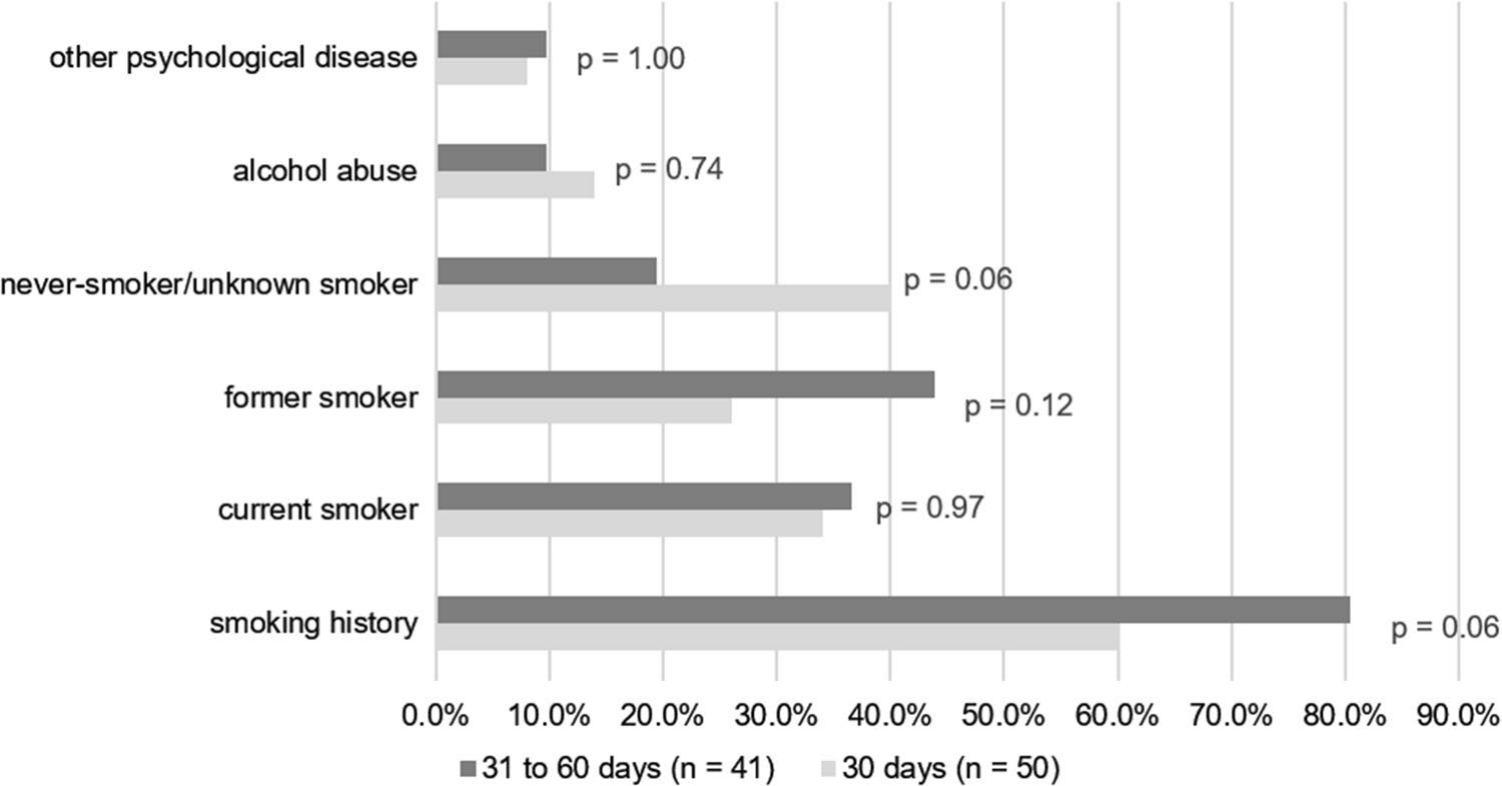
Relative frequency of psychological and behavioral factors stratified by mortality. Legend: P-values from Chi^2^-test

Overall, 69.2% of patients had a history of smoking and 12.1% a history of alcohol abuse, and 8.8% other psychological disease. We did not find significant differences between the two mortality groups regarding these psychological and behavioral factors. Figure 2 shows the distributions of psychological and behavioral factors stratified by mortality.

### Other comorbidities

**Fig. 3:**
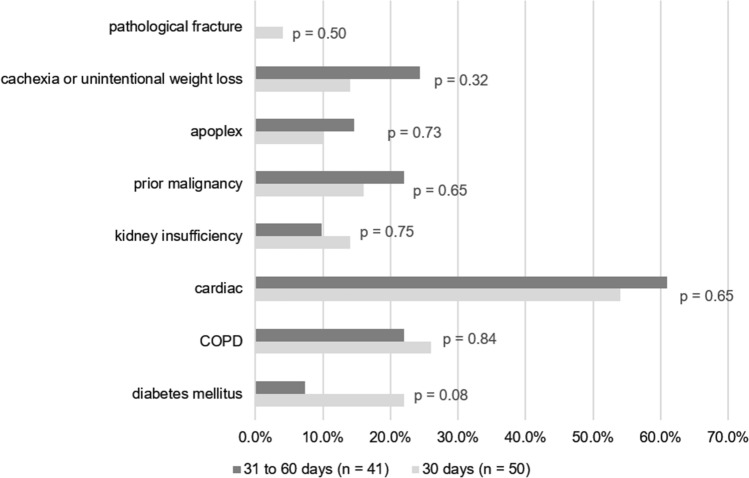
Relative frequency of comorbidities stratified by mortality: Legend: *P*-values from Chi^2^-test. COPD = chronic obstructive pulmonary disease

Patients in the 30-day mortality group more often had a diagnosis of diabetes mellitus (26.0% vs. 22.0%), and renal insufficiency (14% vs. 9.8%); however, the difference with the 60-day mortality group was not significant. The distribution of all comorbidities across the two groups are presented in Fig. 3. BMI did not differ significantly between the groups, although 51.2% of patients in the 60-day mortality group had a BMI over 25 compared to 30.0% in the 30 day-mortality group.

### Subgroup and sensitivity analyses

There was no difference in the group of patients with stage IV at diagnosis and those excluded due to death related to surgical complications compared with the analysis of the entire study population. The comparison of patients in the subgroup of patients who received specialized palliative care resulted in significant differences in the proportion of patients who died unexpectedly (0% vs. 19.3%, p-value = 0.006), and dying in an acute care setting (32.4% vs. 75.4%, p-value = 0.0001) or in a non-acute setting (64.7% vs. 3.5%, *p*-value ≤ 0.0001). 63.0% of patients who died unexpectedly were females, which was significantly (*p*-value = 0.04) more than in all other patients (30%). 27% of patients who died unexpectedly had experienced unintentional weight loss or were cachectic at diagnosis compared to 17.0% of all other patients. This difference was not significant.

## Discussion

In the past decade, the treatment paradigm for advanced lung cancer has evolved dramatically with the advent of immunotherapy and molecularly targeted agents. In light of this progress, evaluating trends in early mortality and predictors thereof is needed. The main aim of our study was to detect factors associated with 30- and 60-day mortality in lung cancer patients. Additionally, in the context of checkpoint inhibitor and TKI treatment our objective was to characterize cause and place of death, as well as previous hospital interaction and comorbidities of patients who died within 30 and 30–60 days of diagnosis. We found that early mortality was significantly associated with older age and more advanced stage at diagnosis. Furthermore, we found that patients in the 30-day mortality group died significantly more often in an acute care setting and were significantly more often treated in the ICU or IMC compared to patients who survived between 30 to 60 days after diagnosis. Further, the prevalence of comorbidities like diabetes mellitus and renal insufficiency was higher in patients dying within 30 days of diagnosis. Surprisingly, 12.0% of patients in both groups died unexpectedly. This group of patients was dominantly female, and a high proportion had experienced unintentional weight loss or were cachectic at the time of diagnosis.

In our cohort, 2.0% of patients died within 30 days, and 5.7% died within 60 days of diagnosis, whereas studies from France (2010), the UK (2000–2013), and the USA (2006–2015) reported 30-day mortalities of 9.7%, 10.0%, and 7.2%, respectively [6, 10, 11]. This difference might be in part explained by the source of the data in the studies. While we reported data of a tertiary hospital, data for the other studies was mostly derived from national databases. While the analysis of large databases allows better generalization of results, we were able to include clinical information not included in these large databases. Additionally, we excluded patients with insufficient follow-up, which can lead to an underestimation of early mortality. Although, we conduct a thorough follow-up for primary cases as part of the clinics certification process, non-primary cases can have an effect on mortality estimates here.

Certain factors associated with early mortality highlighted in our study were previously reported especially, age, male sex, and metastatic disease at diagnosis [3, 6, 11]. However, we did not find a significant association of sex and early mortality in our analysis.

In line with previous studies, the proportion of patients with advanced stage of the disease at diagnosis (stage III or IV), was significantly higher in patients with early mortality. Interestingly, the proportion was higher in the 60-day mortality group compared to the 30-day mortality group, which might indicate therapy-related casualties. An analysis of cause of death strengthens this interpretation as the proportion of patients with complications from therapy and infection was higher in the 30-day mortality group compared the 60-days mortality group. Additionally, patients dying within 30 days were more likely to die in an acute setting, whereas patients dying within 60 days were more likely to die in a non-acute setting, e.g., palliative ward. This implies that a higher proportion of patients in the 60-day mortality group had already made preparations in a palliative treatment situation.

According to the literature, superior vena cava syndrome is an independent predictor of shortened survival in lung cancer [12]. This is in line with findings in our research. Early detection and adequate treatment of superior vena cava syndrome can thus be one key to prolong survival among patients with lung cancer. In general, oncologic emergencies are a significant cause of morbidity and mortality in patients with lung cancer. The knowledge and correct identification of the main oncologic thoracic emergencies of patients with lung cancer therefore enables optimal diagnostic and therapeutic management [13].

Most of the patients with early mortality did not receive any specific cancer treatment, e.g., 62% of patients in the 30-day mortality group did not have a documented tumor-directed therapy. This result is comparable to a French study [6], but lower than a study conducted in the USA where around 70% of patients with 30-day mortality did not receive any treatment [3]. There is a need to improve early diagnosis to give the patients a chance to receive a systemic treatment to reduce time to treatment and then to improve early mortality. Early diagnosis can give the patients a chance to get a systemic treatment before deterioration of the general condition (better PS, early-stage tumor, less weight loss). However, the often long waiting time for the molecular pathology testing could potentially have negative effects. Individual outcome is variable, and even the most favorable patient group contains people with very short, intermediate or long life expectancy. Therefore, identification of predictive features in lung cancer patients deserves further investigation.

An analysis of patients’ comorbidities showed a higher proportion of patients with diabetes mellitus and renal insufficiency in the 30-day mortality group. Patients with lung cancer often have comorbidities, for example diabetes mellitus with a prevalence of 15–17% [14, 15]. In our study, we found that the prevalence was 22% in patients in the 30-day mortality group. Renal insufficiency in lung cancer patients was found to be around 6% in the USA [14, 16], whereas it was 14% in the 30-day mortality group and 12% in the 60-day mortality group. Both diabetes mellitus [17] and renal insufficiency are associated with decreased survival and can limit treatment options in lung cancer [16–18].

As the source of the dataset used in this study was based on secondary data intended for tumor documentation, certain factors that could have an impact on early mortality could not be included in the analysis. Some variables also included missing information, as these were not a focus of the primary documentation. Additionally, all information comes from within the university clinic. However, by adding clinical information from electronic patient records for the early mortality groups, we believe we added important information that provides an insight on factors related to early mortality in lung cancer in Germany. We did not distinguish between NSCLC and SCLC in our study however, in patients with poor prognosis, the main focus is to improve patient’s quality of life regardless of the histological subtype. Providing information on comorbidities and care settings in patients with early mortality enabled detecting factors that can predict early mortality. The limited sample size does not allow generalization of results, but analyses can be amended with future cohorts.

In our study, we were able to detect differences in causes of death between these groups. Additionally, our study looked at factors that have not been analyzed previously such as cause and place of death, as well as comorbidities. Analyzing the cause of death and place of death have only been investigated in a few studies. We found that these two factors provide crucial information in the analysis of risk factors for early mortality. Additionally, as the original dataset was derived from the tumor documentation system the quality of the information in this part of the dataset is exceptionally high.

Our results suggest that we need a greater focus on older and frail patients. Moreover, physicians should pay special attention to females with recent weight loss and patients with a comorbidity of diabetes mellitus or renal impairment. Even if life might not be prolonged, the early integration of palliative care can help patients and families confronted by death in an acute setting and improve quality of life. Engaging a case manager focused on detecting patients with the above characteristics could help improve overall care. Improving survival requires diagnosis at an earlier stage and better organization of diagnosis and specific care pathways.

### Supplementary Information

Below is the link to the electronic supplementary material.

Supplementary file1 (PNG 26 KB)
