# Supplementary figures and images for: Early mortality in German patients with lung cancer: risk factors associated with 30-and 60-day mortality
Source: Clin Exp Med. 2023 Sep 12;23(8):5183–90. doi: 10.1007/s10238-023-01187-x (PMC10725334; doi:10.1007/s10238-023-01187-x)

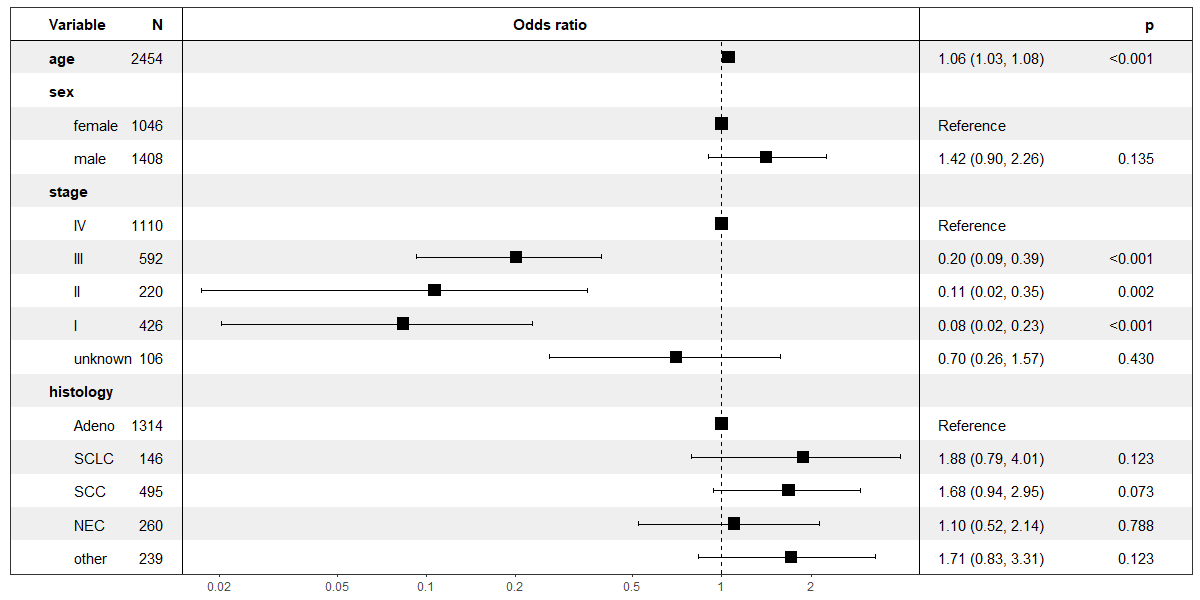

Supplement: Supplementary file 1 — Supplementary file1 (PNG 26 KB) [file 10238_2023_1187_MOESM1_ESM.png]
